# Validity of Functional Performance Outcomes and Gait Metrics Measured by Smartphone Application in Patients with Chronic Stroke

**DOI:** 10.3390/s26216947

**Published:** 2026-11-03

**Authors:** Gabriela Rozanski, Jessica Polizzi, Aidan Rogers, Megha Amin, Wilber Parada Iraheta, Karolina Roszkowski, Jenna Tosto-Mancuso, David Putrino

**Affiliations:** Department of Rehabilitation and Human Performance, Icahn School of Medicine at Mount Sinai, New York, NY 10029, USA

**Keywords:** stroke, functional mobility, performance-based measures, patient-reported outcomes, smartphone, gait analysis, validity

## Abstract

Accurate assessment of impaired functional mobility is crucial to track recovery and guide interventions after stroke. Conventionally, physical therapists administer semi-objective rating scales or manually record performance on various tests. Technological developments, particularly automated algorithms that use motion data from inertial sensors, offer a promising solution for more efficient and objective evaluations. This cross-sectional analysis examined the agreement between therapist-recorded measures and a smartphone application (OneStep) for the Chair Stand Test (CST), Timed Up and Go (TUG), and the Six-Minute Walk Test (6MWT) in 42 participants with chronic stroke (mean age 54 years, 50% male). Analysis was measured via a two-way random-effect intraclass correlation coefficient analysis ICC(2,1) (absolute agreement) and Bland–Altman limits of agreement. ICC(2,1) was 0.989 [0.980, 0.994] for the CST, 0.995 [0.985, 0.998] for the TUG, and 0.921 [0.776, 0.967] for the 6MWT. Mean differences (therapist–app score) were −0.08 repetitions (CST), −1.03 s (TUG). and −69 feet (6MWT). Within-session repeatability was not detectably different between the app and therapist for the CST (SEM ratio app/therapist 0.91 [0.76, 1.05]) or the TUG (1.36 [0.92, 2.13]); the TUG estimates are imprecise and do not establish equivalence. The 6MWT was not considered in within-session repeatability as multiple tests were not completed due to patient fatigue. App failure rates were relatively low for the CST (2/80), TUG (3/76), and 6MWT (5/36). Correlations with patient-reported outcomes, clinician-rated outcomes, and gait metrics were exploratory (89 of 115 significant after false discovery rate correction). The results suggest close agreement for the CST, a small systematic difference for the TUG, and larger differences for the 6MWT. Future research should address the longitudinal predictive utility and broader implementation throughout the health care system of mobile applications like OneStep.

## 1. Introduction

Mobility deficits from neurological injury affect independence, life participation, and overall health. In stroke rehabilitation, the accurate assessment of physical function along the recovery continuum is pivotal to guiding therapeutic interventions and monitoring patient progress [1,2]. Locomotor ability reflects diverse underlying physiology and predicts many different future consequences while influenced by complex external factors [3]. Clinical performance-based outcome measures are key evaluation tools that traditionally rely on therapist judgment after visual observation (semi-objective rating scales) and manual recording (e.g., time, repetitions, distance), which can introduce variability from human error or assessor bias. Expertise is required for appropriate use and interpretation. Advancements in technology offer promising solutions to efficiently enhance the objectivity of labor-intensive test protocols. Researchers have developed sophisticated laboratory techniques for characterizing gait and balance, including wearable sensors, image processing, and floor sensors. These methods utilize equipment such as instrumented walkways, multiple cameras, or specialized sensors, often requiring dedicated laboratory space and highly trained personnel. However, these pieces of equipment are often costly, cumbersome, and create conditions of limited ecological validity [4].

A smartphone-based gait analysis app may provide a more accessible and affordable alternative, while still offering detailed data collection [5,6]. This would be particularly beneficial for clinicians and patients, enabling consistent, detailed, and accurate assessments in a variety of environments, allowing for broader application and integration into existing practice. Unobtrusive, miniaturized movement monitors with online processing and user-friendly interfaces allow much greater feasibility in a clinic setting [7]. Consumer smartphones and their integrated inertial measurement units (IMUs) allow for seamless activity capture, even remotely, without need for additional equipment, providing a solution for gait and balance analysis [8]. Motion data collected may then be analyzed by algorithms, providing a novel approach to assess patients [6]. These systems add precise impairment-level quantitative characteristics, including spatiotemporal deviations, such as decreased cadence, asymmetries in step length, stance, or time, especially for patients with unilateral limb dysfunction [4,6]. Current standard clinical assessments outlined in clinical practice guidelines objectively measure gait speed; however, these spatiotemporal deficits are assessed subjectively [1]. Quantitative data captured by wearable sensors may assist in identifying specific spatiotemporal deficits and elucidate factors underlying performance problems that occur. Subsequently, enhancing clinician decision making allows for more precise targeted intervention selection. Therapists require accessible tools generating well-validated, actionable information for their patients and care teams. Research has shown that mobile applications and wearable inertial sensors are comparable to conventional methods of measuring fall risk factors among older adults [5,9]. Specific kinematic metrics might contribute valuable insights to optimize management of certain conditions, such as multiple sclerosis [10,11]; however, automated test administration using accelerometer data can be complicated by impaired movement patterns [12,13]. Several studies have validated smartphone gait/postural control analysis with healthy participants [14] and more recently in patients [15], including post-stroke individuals [16,17,18]. ‘Instrumented’ versions of Timed Up and Go (TUG) have been explored in various populations [19]. However, while these studies have investigated the validity of IMUs and wearable sensors, they are largely focused on limited metrics or have not compared findings with current clinician assessments. There exists a gap in comparing the primary result obtained by different methodologies (i.e., traditional stopwatch tests versus sensor/algorithm duration), particularly when performing well-validated performance measures for persons with chronic stroke [1]. A digital health device that could both administer standardized clinical measures and collect additional biomechanical data has significant benefits for streamlining assessments with enhanced comprehensiveness. Therefore, this study investigated the agreement between an FDA-registered smartphone application utilizing a built-in IMU to measure acceleration and orientation (OneStep; Celloscope Ltd., Tel Aviv, Israel) and a clinical standard physical therapist assessment when evaluating functional performance outcomes in patients with chronic stroke. Secondary aims were to examine within-session repeatability of the CST and TUG, as well as convergent (construct) validity through testing exploratory associations between app-derived performance measures, PROs, and spatiotemporal gait parameters.

## 2. Materials and Methods

### 2.1. Experiment Design

This was a cross-sectional analysis of data collected from a convenience sample of patients with chronic stroke attending the Abilities Research Center, an advanced technology outpatient clinic at the Mount Sinai Hospital in New York, NY, USA, between October 2023 and May 2024. In compliance with the Declaration of Helsinki, all subjects provided informed consent through a signed form approved by the Mount Sinai Program for Protection of Human Subjects Institutional Review Board (STUDY-23-00276).

### 2.2. Participants

Participants were eligible for inclusion based on the following criteria: (1) age over 22 years old, (2) greater than three months since cerebrovascular accident, (3) symptoms of arm weakness and/or difficulty with balance, and (4) ability to follow commands. Subject records where functional performance outcomes were unable to be obtained (unsafe or unable to stand for any mobility tests) were excluded.

### 2.3. Assessment Protocol

Participants underwent a one-time assessment battery for enrollment in a neurorehabilitation program for patients with chronic stroke. As part of the assessment, patients completed patient-reported outcome (PRO) measures and functional outcome measures. Outcomes were collected in the following order: Modified Rankin Scale (mRS), Barthel Index, Berg Balance Scale (BBS), Chair Stand Test (CST), Timed Up and Go (TUG), 10-Meter Walk Test (10MWT), Functional Gait Assessment (FGA), and 6-Minute Walk Test (6MWT). Between trials and between tests, participants were given a 1–5 min seated rest break as needed for fatigue.

Participants performed one trial of the 6MWT and two trials each of the CST and TUG, respectively, with a OneStep-enabled smartphone secured on the non-hemiparetic limb in their pants pocket. If participants were wearing clothing without suitable pockets, the smartphone was secured in an external pouch with a strap that maintains its position against the thigh. The external pouch consisted of a top strap secured around the participant’s waist, and a lower strap secured around the thigh. Each test was manually initiated in the application by the therapist, and participants were instructed to begin the test upon the audible ‘Go’ signal from the app following a 10-s countdown. Simultaneously, therapists completed manual assessment and measurement consistent with Academy of Neurologic Physical Therapy (ANPT) Core Outcome Measures Clinical Practice Guideline (CPG) recommendations and Knowledge Translation Task Force standardized administration protocols (counting repetitions for CST, timing TUG with a stopwatch, measuring distance ambulated during 6MWT) [1,2]. At the end of the test, the app produced an auditory ‘recording stopped’ for the CST and 6MWT after the respective 30-s or 6-min test duration. For TUG, once the participant was seated, the therapist performed a swipe on the smartphone, which indicated the end of the recording window. During testing, participants were permitted to utilize a customary gait aid (e.g., cane, walker) and/or an assistive device (e.g., ankle–foot orthosis) but ambulated without physical assistance from another person. If participants were unable to perform sitting to stand without upper extremity support, use of armrests was permitted during the CST and TUG.

The (30-s) Chair Stand Test (CST) was designed to assess lower body strength as part of the Fullerton Functional Fitness Battery [20]. It has demonstrated excellent reliability in acute stroke [21] and can avoid potential floor effects associated with timed tests [22]. Subjects were instructed to perform full repetitions from a standard chair (18 inches high) positioned against the wall for 30 s upon a ‘Go’ signal (with more than halfway up at the end counted as a full repetition) [20]. A practice trial was offered, and use of armrests was permitted as necessary [23].

Importantly, the CST has demonstrated construct validity in stroke populations, showing improved performance is associated with improved measures of mobility, balance, and low extremity motor function, including the TUG and Stroke Impact Scale (SIS) [22]. Furthermore, sit-to-stand performance has been independently validated in individuals with chronic stroke, demonstrating strong relationships with muscle strength, balance, and gait [23]. Given that the CST captures repetitive sit-to-stand ability, it similarly reflects a key functional task frequently impaired after stroke. Together, these studies [20,21,22,23,24,25,26,27] support its validity as a measure of functional lower extremity performance in chronic stroke populations.

TUG is a valid and reliable test for quantifying functional mobility in chronic stroke [26,27], with the ability to predict falls among community-dwelling older adults [28,29]. Subjects were given the following instructions: on ‘Go’ (time starts), stand then walk to a floor marker three meters away, turn around, and return and sit (time stops) as quickly and safely as possible [30].

The Six-Minute Walk Test (6MWT) evaluates endurance and aerobic capacity with excellent psychometric properties across multiple patient populations, including chronic stroke [31]. The study space consisted of a 40-foot-long flat, clear pathway marked at each end to standardize administration as recommended by the ANPT CPG [1]. Participants were instructed to ambulate as many 40-foot laps as possible in 6 min, performing ambulatory turns at each end of the walkway. Participants were allowed standing rest breaks as needed. Self-paced laps were counted, and total distance (in feet) was calculated. A manual measurement was performed to determine the distance ambulated during the final incomplete lap.

As part of the clinical evaluation, additional outcomes were assessed. The modified Rankin Scale (mRS), Barthel Index, and National Institutes of Health Stroke Scale (NIHSS) were used to assess disability, functional independence, and stroke severity, respectively. Additional performance outcomes were collected by the therapist for evaluating mobility, also in accordance with ANPT Core Outcome Measures CPG recommendations and Knowledge Translation Task Force standardized administration protocols [1,2]. The Berg Balance Scale (BBS) assesses static and dynamic balance with 14 items of varying difficulty scored on a 0–4 scale determined by the participant’s ability to perform each activity. The Five Times Sit to Stand (5xSTS) assesses transfer ability, with participants instructed to stand up and sit down from a chair five times in a row as quickly as possible. The therapist records the amount of time taken to complete the repetitions. The 10-Meter Walk Test (10mWT) is a gait speed assessment during which a participant is instructed to ambulate a 10-m distance first at a comfortable speed for two trials and then at a fast pace for two trials. The therapist records the time taken to ambulate the middle six meters. The average of the two trials for comfortable and fast gait speed, respectively, is recorded [32]. Functional Gait Assessment (FGA) is a 10-item assessment of walking balance and ability to perform multiple motor tasks while walking, scored on a 0–3 scale based on performance [33].

Patient-reported outcomes were collected via surveys sent by email, including the Stroke Impact Scale (summed ADLs and mobility domains; last question rating subjective level of recovery on a scale from 0, none, to 100%, full, SIS-9) and EuroQol-5D-5L (overall health status quantified on a 0, worst, to 100, best visual analog scale (VAS), severity of problems with mobility and usual activities dimensions rated from 1, least, to 5, most).

### 2.4. Data Processing

Data were collected via the OneStep application installed on an iPhone 13 (iOS 17.0.3–17.4.1). Gait was measured with OneStep’s smartphone gait analysis system (processing algorithm versions [2.18–4.0]; Celloscope Ltd., Tel Aviv, Israel). The system operates on both iOS and Android devices; raw motion sensor data are collected in a standardized manner, and the gait analysis algorithm is platform-agnostic. The smartphone movement was recorded at a sampling rate of 100 Hz using the standard integrated inertial measurement unit (IMU) that includes accelerometers and gyroscopes. For the CST, alerts can be heard to indicate when 20, 15, and 10 s remain preceding a five-second countdown until the end. Motion data uploaded to the application’s servers was analyzed after it was projected into the perceived plane of movement through a peak-finding algorithm analyzing rotations on the horizontal axis for detecting sit-to-stand events for 30 s from ‘Go’. TUG duration was derived from the detection of the sit-to-stand and final stand-to-sit transitions in the smartphone’s inertial signal, independent of the timing of the manual start and stop commands performed by the therapist, which serve only to define the recording window. Patterns corresponding with each segment of the TUG (i.e., rise, walk to marker, turn around, and return and sit) were extracted to measure the time elapsed for each event and total test duration. Inertial motion data collected during the 6MWT were segmented into individual gait cycles, and any unrelated noise/artifact (e.g., static postures, phone shaking) was filtered out. A previously validated [34,35,36,37] deep learning framework calculated the following spatiotemporal parameters: cadence; velocity; variability (normalized to cadence/velocity); base width; double support; and step and stride lengths. Asymmetry metrics are reported as the absolute magnitude of percent difference between sides (relative to stride for step length asymmetry). Consistency measures, on a scale from 0 to 100, how similar strides are to one another. The walk score is a proprietary composite that summarizes the subject’s gait quality, integrating velocity, cadence, base width, double support, hip flexion range, and stance/step length asymmetry. The total distance walked in six minutes (between ‘Go’ and ‘Recording stopped’ signals) is obtained from the above variables, primarily stride lengths, while also incorporating turns. After analysis, each test result was displayed in the application and accessible through a web-based clinical dashboard.

Values analyzed for each participant’s CST and TUG were averaged across trials in which both the therapist and the app produced a valid value; participants with only one matched valid trial contributed only that trial to the analysis. An app trial was classified as a failure when the application reported an error and no values were imputed. Participant- and trial-level accounting is reported with explicit denominators (Results 3.1). All results, other than app-reported failures, were retained in the primary analysis; outliers were examined in a sensitivity analysis (Section 2.5) aside from one app CST trial (participant 21, trial 2), which was excluded before analysis because the phone was not secured in the strap. This was a procedural exclusion and not an app failure. The therapist did not record a trial for this participant, so the exclusion did not change the paired analysis (*n* = 77, CST pairs). Outliers were retained and are reported separately.

### 2.5. Statistical Analysis

All data collected by the therapist were stored in a web-based REDCap (Research Electronic Data Capture, version 13.1-14.0) project securely hosted at the Icahn School of Medicine at Mount Sinai. Analyses were performed using Python (version 3; pandas, SciPy, and statsmodels). Descriptive statistics are presented as count (percentage) and mean (standard deviation) or median (range) based on the distribution of the quantitative data type, examined for normality with Shapiro–Wilk tests; most data were non-parametric.

Agreement between therapist- and smartphone-measured functional performance outcomes was quantified with intraclass correlation coefficients (ICCs) from a two-way ANOVA decomposition, with participants’ measurement method as random effects. The primary agreement analysis compares, for each participant, a single therapist value against a single app value per test. For the CST and TUG, that is the average app vs. therapist score for matched valid trials (only trials where a participant has both a therapist and app measurement from the same session, up to two trials total). For the 6MWT, only one trial was administered per participant to avoid fatigue. The primary statistic used was an ICC(2,1) because it measures the absolute agreement between two methods applied to the same participants (therapist vs. app) and accounts for systematic bias (mean differences) between said methods [38,39]. In this study, the ICC(2,1) is computed and reported using the conventional ‘two-way random-effects absolute agreement’ formula [39]. Every participant was assessed by both the app and therapist, but this does not justify treating the ‘method’ as a random factor from a random population of interchangeable methods. Therefore, the intended estimand of this analysis is the agreement between the specific therapist-administered assessment and the one-step app; however, given this sample, findings should not be interpreted as evidence that the app would agree similarly with a different rater, different version of the app, or different measurement device. Two corroborating ICC analyses were reported, one-way random effects ICC(1,1) and two-way fixed effects ICC(3,1), the latter of which measures the ranking consistency independent of mean differences between methods. All three ICCs are computed from the identical set of paired therapist–app observations. Agreement among the three values, therefore, reflects internal consistency of the dataset under alternative modeling assumptions, not independent validation from a separate sample or measurement occasion; thus, these are corroborating, same-data sensitivity analyses, and a gap between ICC(3,1) and ICC(2,1) would reveal the presence and magnitude of a systematic bias. For each ICC, 95% confidence intervals (CIs) were calculated and confirmed with participant-level percentile bootstrap intervals (2000 resamples) as an assessment of robustness. Each participant was assessed by one therapist; thus, the ICC does not accurately estimate inter-rater reliability but rather method agreement. To assess whether method agreement varied by therapist, differences (therapist—app) were compared across the four raters using the Kruskal–Wallis test and visualized using Bland–Altman plots [40,41]. Sensitivity analyses included: (1) ICC computed for the primary therapist (therapist one, *n* = 31) alone, (2) leave-one-out analyses in which ICC was recomputed after removing each therapist in turn, and (3) leave-one-out analyses in which ICC was recomputed after removing each participant in turn. These tests evaluated whether the primary result depended on any single rater. Bland–Altman plots are used to characterize the disagreement between methods through the visualization of each participant’s difference (therapist—app) against their mean score. Plots also report on the mean difference and 95% limits of agreement (mean +/− 1.96 SD).

To determine whether disagreement between methods varied systematically with measurement size, linear regression and Spearman correlation of absolute differences with the pair mean were performed to identify heteroscedasticity and signed differences to identify proportional bias. For positive-valued measurements (6MWT distance), ratio limits of agreement (LoA) were computed using log-transformed data (to account for measurement error scaling with measurement magnitude). Additionally, non-parametric Wilcoxon signed-rank tests were used to further assess bias in non-normally distributed data (CST, TUG), whereas paired T-tests were applied to normally distributed data (6MWT). Agreement was interpreted against literature-based thresholds: minimal detectable change (MDC) values of 2 repetitions for the CST, 3.2s for TUG, and 13% smallest real difference (SRD%, relative to the mean) for the 6MWT [21,42,43]. MDC and SRD are used here as a measurement error threshold as opposed to a judgement of clinical importance. The 6MWT percent difference is a variable threshold that scales with measurement magnitude, which is reflected in the figures. Differences were compared across therapists (Kruskal–Wallis test).

The CST and TUG were performed twice per participant, and the 6MWT only once. To assess within-session repeatability for each participant with two valid matched trials, this work reports ICC(2,1), ICC(1,1), and ICC(3,1) with its standard error measurement (SEM = $\sqrt{\left(\mathrm{MSE}+(\mathrm{MSC}-\mathrm{MSE})/n\right)}$), the minimal detectable change (MDC95 = 1.96 × $\sqrt{2\times \mathrm{SEM}}$), and bootstrapped confidence intervals (trial 2—trial 1) [44].

As part of exploratory analyses, outliers were examined via Tukey’s fences (1.5 interquartile range 1.5; extreme beyond 3) applied to paired differences. Analyses were repeated for valid data, data excluding flagged outliers, data excluding extreme outliers in Supplementary Tables, and an additional analysis with the removal of subjects 6 and 33 who performed the task trials with the smartphone attached via strap. Due to the nature of this analysis, as outliers are removed, agreement between measures increases; thus, this analysis is a measure of sensitivity. Additionally, participants with a paired vs. unpaired 6MWT were compared with a Mann–Whitney U test and Fisher’s exact test.

Participants were classified as at increased fall risk from the therapist and the app value using the literature thresholds (6MWT < 331.65 m [45]; TUG ≥ 14 s [46]; CST age- and sex-specific norms [47], available for participants aged 60–94 years). Paired classification agreement is reported as percent agreement, Cohen’s kappa, and prevalence-adjusted bias-adjusted kappa (PABAK). Kappa 95% CIs are reported analytically and by a percentile bootstrap (2000 resamples, seed = 42). These tests describe agreement between two measurements against a threshold; however, it is not validation against actual falls, which were not recorded.

Spearman’s ρ and Pearson’s r correlation coefficients were calculated as measures of associations among gait metrics, patient-reported outcomes, and functional performance measures, along with 95% confidence intervals, using the Fisher transformation with the Bonett–Wright variance [48]. All correlation analyses were treated as exploratory due to lack of pre-established hypotheses; however, all hypothesis tests were two-tailed, and the Benjamini–Hochberg false discovery rate (BHFDR) (q < 0.05) was applied within each table as the primary correction with Holm’s procedure and Benjamini–Hochberg across the same tables as sensitivity analyses [49,50]. Relationship strength was determined according to Cohen’s standard guidelines (0.2, 0.5, 0.8 or 0.1, 0.3, 0.5 for small, medium, and large effects, respectively) [51]. Because app-derived 6MWT distance is calculated from gait variables (i.e., stride length and velocity), associations between the app 6MWT and gait metrics are not reported and were excluded from all multiple-comparison adjustments.

## 3. Results

### 3.1. Participants/Sample Characteristics

There were 42 patients included in this analysis (five subjects omitted according to exclusion criterion) (Table 1) with an equal number of males and females, on average middle-aged (54 +/− 13.2 years old) and overweight (BMI 26 kg/m^2^), large range in chronicity (mean: 2.5 years; range: 0.3–19 years since cerebrovascular accident), and balanced representation between stroke type (ischemic 52% vs. hemorrhagic 43%, missing 5%; left side 52% vs. right side affected 48%). Overall, subjects reported feeling less than halfway recovered (based on SIS-9), a medium level of health (EQ-5D-5L VAS), and moderate problems with ADLs/mobility (SIS/EQ-5D-5L domains). Level of disability (mRS) ranged from not significant to moderately severe (requiring assistance for self-care and ambulation), but performance of daily activities (Barthel Index) skewed high. Median residual impairment due to stroke (NIHSS) was minor. Functional test results (BBS, 5xSTS, 10mWT, FGA) were generally below normative values [52,53,54], indicating limited mobility and possible fall risk for some individuals.

Data from four evaluating therapists were included in this analysis (therapist one, *n* = 31; therapist two, *n* = 5; therapist three, *n* = 4; therapist four, *n* = 2). Participants were assessed by one therapist. Participant and trial accounting are summarized in Table 2. Trial attempts varied by assessment: 80/84 for the CST, 76/84 for the TUG, and 36/42 for the 6MWT (Figure S1). Paired therapist and app values were collected for 42 participants, but the total number of participants with paired tests varied by assessment (CST, 42; TUG, 39; 6MWT, 31) and resulted in 77 (CST), 73 (TUG), and 31 (6MWT) valid pairs (Table 2). The CST and TUG results were averaged (not summed) over the trials with a valid therapist and app value: 35 (CST) and 34 (TUG) participants had two such trials, and seven and five participants had a single valid trial, which was used as their value. The 6MWT was performed once and thus could not be averaged.

App analysis failures indicated by the application occurred in 2/80 and 3/76 trials for the CST and TUG (2.6% and 3.9% error rates), respectively (Table 2). In five 6MWT cases (at least once likely due to baggy pockets not sufficiently stabilizing the smartphone), the application incompletely measured distance but calculated gait parameters (total error rate 13.9%). A possible technical issue is that the stop button could be pressed if the screen does not turn off in a subject’s pocket. One CST trial was omitted from the study sample because of poor procedure (OneStep strap improperly secured, causing unstable/inaccurate smartphone movement detection); however, a therapist did not record a trial for this participant, so the exclusion did not change the paired analysis. The outlier TUG record that measured 17.5 s higher in the application revealed a bug in the current algorithm (difficulty handling prolonged time between test completion and ‘Stop’ button activation) to be fixed for future versions. The application captured additional motion artifacts during the prolonged recording window (i.e., patient adjusting position in chair or handling the smartphone), which led to the algorithm misidentifying the end of the task, lengthening the measured duration. Three CST outliers were identified and further investigated via plots of pitch (hip flexion) angle from the smartphone data: the four count discrepancy was attributed to the application detecting unsuccessful stand attempts (relatively small range, shorter duration movement); two additional repetitions were included for another trial (one partial then the last rising, which the therapist likely deemed less than halfway complete at 30 s); an early first and late final stand appear to have been counted by the therapist in one case. The 6MWT outliers resulted from overestimation of stride length and irregular turning patterns that could not be fully analyzed.

Participants with a paired 6MWT (*n* = 31) and those without (*n* = 11) did not differ detectably in age, time since stroke, mRS, BBS, FGA, comfortable gait speed, 5xSTS, TUG, CST, or sex (smallest *p* = 0.097) across 11 comparisons (Table S6). However, with only 11 participants in the comparison group, these tests are underpowered, so non-significance does not demonstrate equivalence between the two groups.

### 3.2. Primary Results

**Figure 1:**
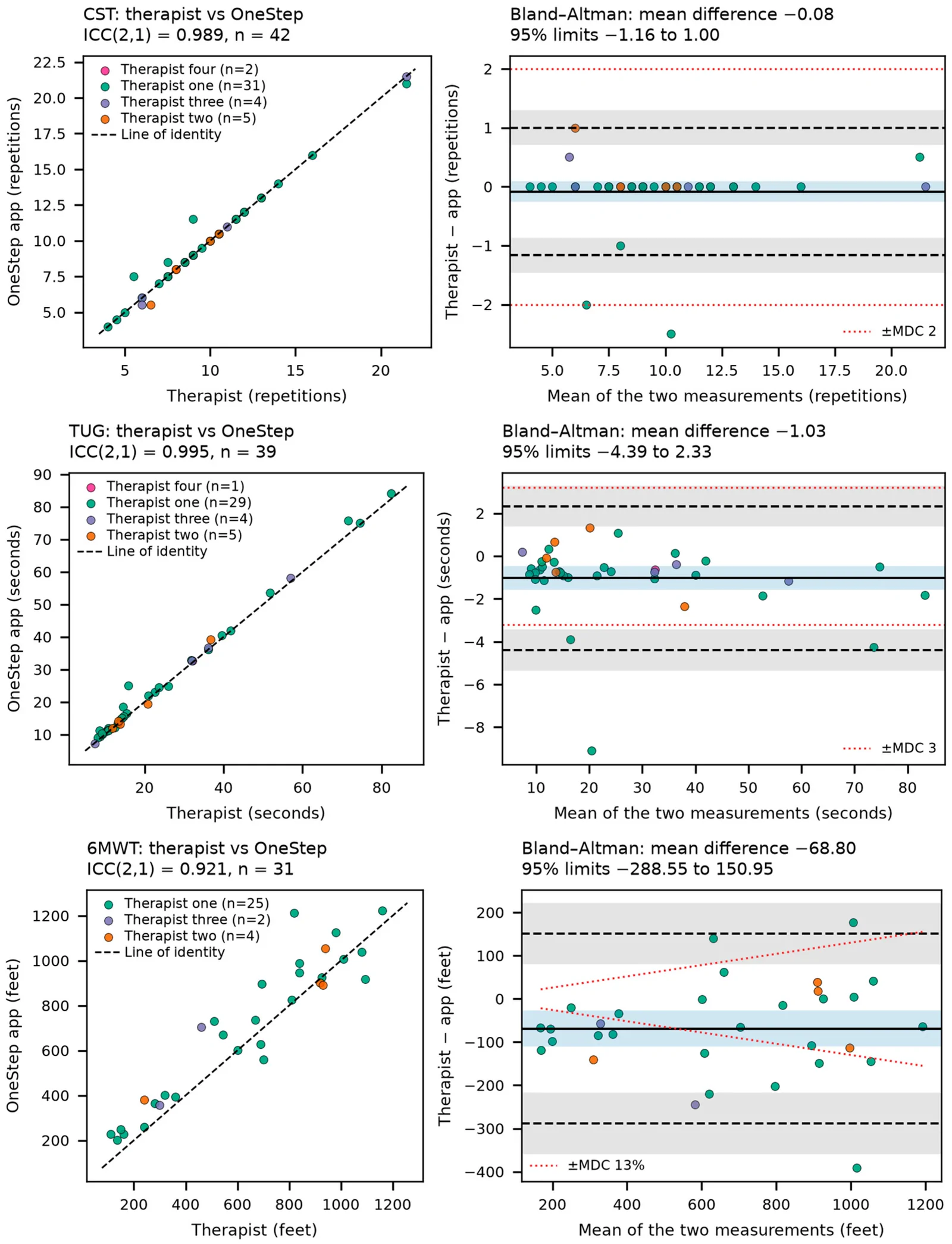
Bland–Altman and identity (correlation) plots of the agreement between therapist- and smartphone application-measured functional performance outcomes (**top**: Chair Stand Test; **middle**: Timed Up and Go; **bottom**: Six-Minute Walk Test). **Left**: identity plots colored by therapist with the line of identity. **Right**: Bland–Altman plots (difference = therapist—app); the solid line is the mean difference, dashed lines the 95% limits of agreement (+/− 1.96 SD), shading the 95% confidence intervals of the mean difference (blue) and of the limits (gray), and red dotted lines the literature threshold (MDC for the CST and TUG, 13% SRD, proportional to the mean for the 6MWT).

Agreement between functional performance outcome measurements obtained from therapist and smartphone application was significantly correlated with large effect sizes and quantified with the intraclass correlation coefficient and Bland–Altman analyses (Table 3, Figure 1). The ICC(2,1) was 0.989 [0.980, 0994] for the CST (*n* = 42), 0.995 [0.985, 0.998] for TUG (*n* = 39), and 0.921 [0.776, 0.967] for the 6MWT (*n* = 31). The one-way ICC(1,1) and ICC(3,1) provided similar results (Table S5). The mean difference between results (CST: −0.08 ± 0.55 (repetitions), TUG: −1.03 ± 1.72 (s), and 6MWT: −68.80 ± 112.12 (ft)) was highest and most variable for the 6MWT; on average, the app recorded greater distances (denoted as negative) and longer TUG times in comparison to the therapist’s recordings. Systematic bias was assessed with a Wilcoxon signed-rank test (CST, *p* = 0.399; TUG, *p* < 0.001) or a paired T-test (6MWT, *p* = 0.002), depending on normality. No test showed proportional bias: *p* = 0.608 (CST), *p* = 0.264 (TUG), and *p* = 0.708 (6MWT). To represent correlations or associations between measures, Pearson’s r (CST: 0.989; TUG: 0.996; 6MWT: 0.940) and Spearman’s ρ were calculated (CST: 0.980; TUG: 0.990; 6MWT: 0.926) (Table S5).

The Bland–Altman plots (Figure 1) show the size of the individual-level differences (units defined for each test) when comparing the app- vs. therapist-measured outcomes. The CST 95% LoA were –1.16 [−1.46, −0.87] to 1.00 [0.70, 1.29] repetitions, relative to the literature MDC (at ± 2.0 reps) [21]. The majority of participants were within said bounds (41/42; 98%) [21]. The TUG 95% LoA was −4.39 [−5.35, −3.44] to 2.33 [1.37, 3.29] with a mean difference of −1.03 s [−1.59, −0.47], which, relative to the literature (± 3.2 s), means 36/39 (92%) participants differed by no more than the threshold and the LoA [42]. The 6MWT LOA was –288.55 to 150.95 ft (−88 to 46 m), with a mean difference of –69 ft (−21 m; −10.9% of the mean therapist distance). Relative to the literature SRD (± 13%), 14/31 (45%) of participants differed by less than said threshold and the LoA beyond it [43].

**Figure 2:**
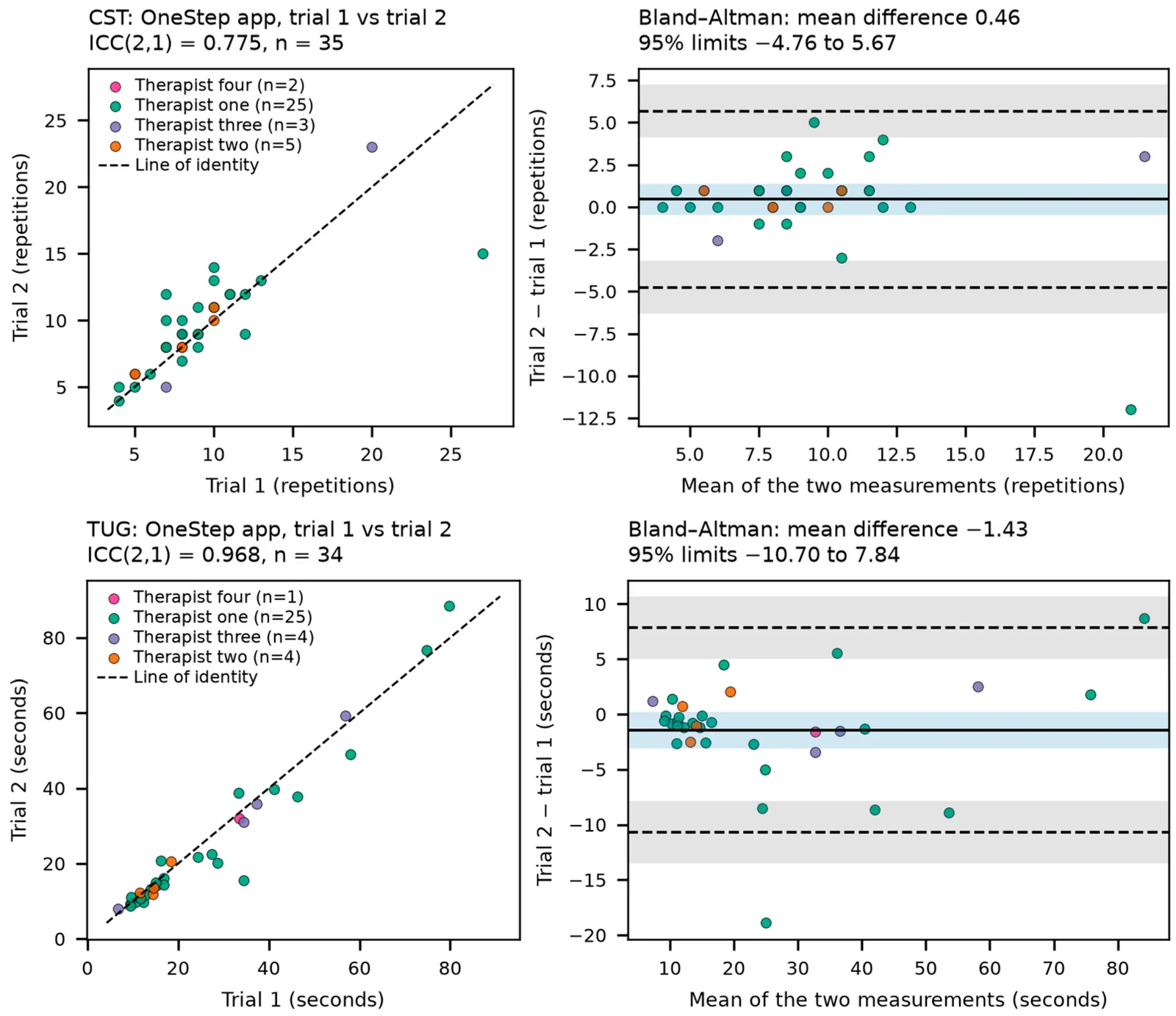
Bland–Altman and identity plots of the within-session repeatability of the smartphone application-measured functional performance outcomes (**top**: Chair Stand Test; **bottom**: Timed Up and Go; trial 2 vs. trial 1, same session). Difference = trial 2—trial 1; lines and shading as in Figure 1.

To assess within-session repeatability for measures where participants completed two trials in the same session (with a seated rest between trials), ICC(2,1) was computed for the two trials of the smartphone-measured CST (*n* = 35) and TUG (*n* = 34) (Table 4, Figure 2; therapist data Figure S2). ICC(2,1) was 0.775 [0.600, 0.879] for the CST and 0.968 [0.936, 0.984] for TUG. Spearman correlation coefficients for these tests (used as a measure of association) were 0.837 (CST) and 0.96 (TUG). The standard error of measurement was 1.88 repetitions (CST) and 3.45 s (TUG), corresponding to a minimal detectable change (MDC95) of 5.22 [2.69, 7.82] repetitions (56% of the mean) and 9.55 [5.61, 13.36] s (38% of the mean). The 95% limits of agreement between trials were −4.76 to 5.67 repetitions (CST) and −10.70 to 7.84 s (TUG). Because within-session TUG errors scaled with duration (Figure S5), ratio limits were used for the app versus therapist comparison (trial 2/trial 1: 0.93, 0.66–1.33) due to heteroscedasticity. To determine the use of the ratio of limits, the absolute trial-to-trial difference against the pair mean for each participant was calculated. The Spearman correlation between the two was ρ = 0.66 (*p* < 0.001) for the TUG app and ρ = 0.75 (*p* < 0.001) for the TUG therapist, and ρ = 0.33 (*p* = 0.050) for the CST app and ρ = 0.29 (*p* = 0.095) for the CST therapist (Figure S5, Table 4). As previously stated, the TUG association is significant for both raters, supporting ratio limits; the CST association is borderline, so absolute limits are retained for the CST. On average, the CST count was 0.46 repetitions higher on the second trial (*p* = 0.020) and the TUG 1.43 s shorter (*p* = 0.054) (Table 4, trial order test column; Figure 2 middle panel, solid bias line). Two trials of the 6MWT were not completed due to patient fatigue; thus, test–test repeatability could not be assessed for this test.

For the same participants, the therapist’s measurements were similarly repeatable and reported in Table 4 (ICC(2,1) 0.740 for the CST and 0.982 for TUG). For the CST, absolute trial-to-trial error was comparable: SEM was 1.88 repetitions for the app and 2.07 for the therapist, giving an MDC95 of 5.2 and 5.7 repetitions (56% and 62% of the mean). For TUG, the app’s SEM was larger at 3.45 s compared with 2.53 s for the therapist, giving an MDC95 of 9.6 s (38% of the mean) versus 7.0 s (29% of the mean). The app was not detectably less repeatable than the therapist: the difference in ICC(2,1) (app—therapist) was 0.035 [−0.015, 0.130] for the CST and −0.014 [−0.077, 0.004] for TUG, and the ratio of SEMs (app/therapist) was 0.91 [0.76, 1.05] for the CST and 1.36 [0.92, 2.13] for TUG. Because the TUG interval includes 1 but extends to about 2, these data do not establish equivalence between methods. The MDC95 values were 2.6–2.9 times the literature CST MDC of two repetitions and 2.2–3.0 times the literature TUG MDC of 3.2 s (Table S5).

Outlier sensitivity and device placement analyses are summarized in Table S2 and Figure S3. Agreement between app and therapist remained even after leaving out any single participant (leave-one-out ICC(2,1) range: CST 0.989–0.994, TUG 0.995–0.999, 6MWT 0.921–0.941). The same pattern can be found throughout the analysis, as expected from removing outliers discerned from differences: ICC(2,1) 0.989 to 0.994 for the CST (two flagged), 0.995 to 0.999 for TUG (seven flagged), and 0.921 to 0.941 for the 6MWT (one flagged). Within-session repeatability proved even more sensitive to these changes. Excluding flagged participants raised the CST ICC(2,1) from 0.775 to 0.927 for the app (eight flagged) and from 0.740 to 0.927 for the therapist and lowered the TUG MDC95 from 9.6 to 6.7 s (app) and 7.0 to 6.2 s (therapist). These changes demonstrate that a minority of participants with large between-trial changes drive the repeatability limits for both raters in this study. Removing these outliers also changed the false discovery rate conclusion of 13 of 115 correlations and reversed the sign of one (Table S3). Device placement was not standardized, as two participants (records 6 and 33) wore the recording device on a strap as opposed to in their pocket. Exclusion of these two participants left agreement (0.00–0.005 change in ICC(2,1)) and mean differences (CST: 0.06 reps; TUG: 0.03s; 6MWT: 0.6 ft) essentially unchanged. The false discovery rate conclusion changed to significant for 3/115 correlations when comparing PROs to app-recorded functional outcomes. Given that only two participants used the strap, this analysis cannot estimate a placement effect, and the main conclusions do not depend on these two participants.

Differences did not vary significantly by therapist (Kruskal–Wallis *p* = 0.258, *p* = 0.438 and *p* = 0.653 for the CST, TUG, and 6MWT; Table S1). A complementary leave-one-therapist-out sensitivity analysis dropped all participants assessed by one therapist in turn and then recomputed agreement on the remaining participants (Table S7, Figure S4). Because therapist one assessed the majority of participants (31/42), exclusion of this therapist had the largest effect on precision. For the CST, *n* fell from 42 to 11, and the ICC(2,1) 95% CI widened to 0.997 [0.989, 0.999] from 0.989 in the whole sample. For TUG, *n* fell from 39 to 10, and the ICC(2,1) 95% CI widened to 0.998 [0.992–0.999] from 0.995 in the whole sample. For the 6MWT, only *n* = 6 remained after dropping 31 participants, below the minimum of eight needed to estimate ICC(2,1), so this sensitivity analysis is not reported. Excluding any of the other three therapists, each of whom assessed six or fewer participants, only modestly changed ICC(2,1) by less than 0.007 for all measures (CST 0.000–0.004, TUG 0.000–0.001, 6MWT 0.004–0.006), with CIs overlapping the primary estimate. With reference to published normative values, paired classification agreement is shown in Table S4. Based on the reported cutoff distance for stroke (<331.65 m) [45], the therapist- and app-measured 6MWT classified 29 (94%) and 28 (90%) of 31 participants, respectively, as having increased fall risk. Both methods were in agreement for 28/31 (90.3%) of participants (27 at risk by both, one not at risk by both). Cohen’s kappa was 0.35 [analytic: −0.35, 1.00; bootstrap: −0.08, 1.00]; both ranges include 0, so the result is imprecise and thus requires a larger sample size in further testing. The kappa is low despite the high agreement because prevalence is high (90–94% at risk of fall). For TUG, agreement was 95% at the 14 s cutoff; kappa was 0.89 [analytic: 0.75, 1.00; bootstrap: 0.70, 1.00] [46]. For the CST, all 15 participants (participants only included if aged 60 years or older) were classified as at risk by both methods (100% concordance). Given this concordance, kappa is not estimable. Of the 15 participants above age 60, eight (53%) used an armrest during the CST, and seven did not. All 15 were at risk regardless of armrest use; thus, agreement was complete yet uninformative given that the use of the armrest places the subject at fall risk [47]. These analyses describe agreement between two measurements relative to a threshold; they do not validate either measurement against actual falls, and the TUG cutoffs are derived from populations other than chronic stroke.

### 3.3. Exploratory Associations

The relationship between patient-reported outcomes and clinical evaluation measures is represented in Table 5. After controlling for multiple comparisons, 32/49 associations remained significant (q < 0.05). Based on Spearman coefficient magnitude (large effect size), the Stroke Impact Scale–ADL domain and mRS were most associated with the functional performance tests (ρ > |0.49|, q < 0.05). SIS mobility (ρ > |0.47|) followed as the next most associated with functional performance. EQ-5D-5L mobility was significantly associated with all seven measures, with |ρ| of 0.55–0.63 for the 10mWT-CGS, FGA, TUG, and 6MWT and 0.35–0.48 for the CST, 5xSTS, and BBS. The 6MWT was strongly associated with each patient’s reported outcome measure (|ρ| > 0.5, q < 0.05), except for the EQ-5D-5L VAS for overall health status (ρ = 0.31, not significant). The FGA was also strongly associated with most patient-reported outcome measures (|ρ| > 0.5), excluding the SIS-9, EQ-5D-5L VAS, and usual activities. EQ-5D-5L VAS had no significant association with any measure after correction, and SIS-9 was significantly associated only with the 6MWT. Four associations were significant at *p* < 0.05 before but not after correction: SIS-9 × 10mWT-CGS (ρ = 0.35, *p* = 0.035, q = 0.053); SIS-9 × TUG (app) (ρ = −0.34, *p* = 0.047, q = 0.065); EQ-5D VAS × 5xSTS (ρ = −0.34, *p* = 0.039, q = 0.056); and EQ-5D activities × FGA (ρ = −0.32, *p* = 0.045, q = 0.063).

All the functional performance measures obtained by both therapist and smartphone were highly correlated (|ρ| > 0.5, q < 0.05), except for the CST and 10mWT-CGS (ρ = 0.43, q = 0.006; Table 6); all 12 associations remained significant after correction.

Analysis of motion data from the 6MWTs utilizing previously validated algorithms generated the variables presented in Table 7 for 36 subjects [6]. On average, gait performance was relatively low compared to older adult normative values [55], suggesting limited community ambulation [56] and risk of adverse outcomes [57] for the study sample overall. Most notably, subjects in [55] ranged in age from 70 to 85+, and across all age groupings, they had a mean cadence > 102 (steps/min), double support < 30.3 (%GC), and stride length > 1.11 (0.15) (m). All these measures were measured across a group of over 300 subjects and outperformed our participants’ recorded results. In comparison to results in Table 7, this study’s subjects have a decreased cadence, higher double support time, and shorter stride length.

The association of spatiotemporal parameters with clinical evaluation measures is shown in Table 8 (false discovery rate adjusted within the table: 45 of 54 associations are significant at q < 0.05). Velocity, walk score, double support, stride length, and cadence variability were all strongly associated with the BBS, 10mWT-CGS, FGA, and TUG (|ρ|: 0.74–0.90, q < 0.05). Associations with the 30s CST (|ρ|: 0.40–0.46) and 5xSTS (|ρ|: 0.34–0.48) were weaker, and stride length vs. 5xSTS (ρ = −0.34) was not significant after correction (q = 0.052). The BBS and FGA were strongly associated (*p* < 0.001, ρ > |0.5|), with each spatiotemporal parameter except stance and step length asymmetries. A similar pattern of large effect sizes could be observed for the 10mWT (comfortable gait speed, CGS) and TUG to a lesser degree. Both tests showed weaker associations (ρ = −0.41 and 0.37 for the 10mWT-CGS; 0.42 and −0.44 for TUG), whereas stance and double support asymmetry were moderately to strongly associated with both (|ρ|: 0.51–0.79).

## 4. Discussion

These cross-sectional findings suggest close agreement between therapist and smartphone application (OneStep) measurements of the CST and TUG (with small systematic differences) along with larger systematic and individual-level differences for the 6MWT. Regarding the agreement between measurement modalities, ICC(2,1) values were high (0.921–0.995; Table 3), and app error rates were low (CST 2/80 trials, TUG 3/76 trials, 6MWT 5/36 tests). However, a high correlation or ICC alone does not inherently establish the interchangeability of two methods. Intra-class correlation coefficients penalize systematic bias (unlike classic correlation analyses, i.e., Spearman) since it is determined by the ratio of between-participant and total variance, but heterogeneous samples can also skew the ICC score. As an example, the 6MWT ICC(2,1) was 0.921, yet the actual limits of agreement (−88 to 46 m) were wider than the 13% SRD threshold (expressed as a percentage of the mean, −43% to 23%), leaving over half of participants (17/31) with differences between measures beyond the SRD [43] (Figure 1). However, without an independent distance reference, it is unclear if this discrepancy is due to therapist underestimation or instrument error. Moreover, analysis of Table 3 reveals that systematic bias was present for the TUG and 6MWT (*p* < 0.05), implying significant differences between therapist and app measurements. Only the CST demonstrated close agreement with therapist measurement (ICC = 0.989, *p* = 0.399, no significant bias), whereas the TUG and 6MWT showed good validity but may require calibration correction.

Multiple exploratory associations were identified between outcomes and other recorded measures. Smartphone-based gait analysis was consistently completed during the 6MWT; the spatiotemporal parameters extracted (in particular, velocity, walk score, stride length, double support, and cadence variability) and performance outcomes (BBS, FGA, and 6MWT) were strongly associated with one another (ρ > |0.5|, q < 0.05). Strong associations also exist between PROs and app-recorded standard functional assessments (CST, TUG, and 6MWT) and some spatiotemporal parameters (Table 5). Specifically, subjective reports of disability (mRS) and difficulty/problems with mobility (SIS mobility and EQ-5D-5L mobility) or daily activities (SIS-ADLs) were associated (ρ > |0.48|, q < 0.05) across one or more of these three different clinical tests. The 6MWT showed additional correlations with perceived recovery (SIS-9) and usual activity performance (EQ-5D-5L usual activities) in this sample (ρ> |0.5|, q ≤ 0.05) (Table 5). However, the EQ-5D-5L, SIS-mobility, and SIS-9 did not have strong correlations with the CST, and the SIS-9 and SIS mobility did not have a strong correlation with TUG (ρ < |0.5|). No significant relationships were found with overall health status (EQ-5D-5L VAS), suggesting that this is a separate construct (Table 3). Previous work has shown locomotor ability, including walking speed, predicts life participation after stroke [58], and that other capacity-based measures may be important determinants as well. This study’s exploratory associations between PROs and functional assessments are consistent with these findings.

Future work should explore the addition of objective spatiotemporal gait assessment to enhance objectivity of physical assessment. Given that almost all therapist- and smartphone-measured outcomes featured strong associations (ρ > |0.5|, q < 0.05) (Table 6), it is plausible that spatiotemporal measurements collected by a smartphone application could enhance objectivity of assessment and identification of impairments. The multi-item, ordinal-scored BBS and FGA have possible observer bias and floor or ceiling effects [59,60] compared to objective, continuous variable tests. Previous work has demonstrated large effect size associations between these scales and many spatiotemporal parameters [61,62]. This work corroborates these findings and demonstrates that 8/9 spatiotemporal measurements are highly correlated with the BBS (ρ ≥ |0.5|, q < 0.05) and 7/9 with the FGA (ρ ≥ |0.5|, q < 0.001) (Table 6). These same spatiotemporal measures (walk score, double support, velocity, cadence variability, and stride length) that are strongly associated (ρ > |0.74|, q < 0.05) with at least one performance measure are also correlated with other functional performance measures (excluding the 5xSTS and CST). Correlation of these exams may enhance practitioners’ ability to assess and identify deficits associated with functional balance tasks assessed on the FGA and BBS, providing a more robust capture of patient performance with potential to more objectively measure and track progression over time. Future work should look to create larger databases of this patient population‘s spatiotemporal parameters to enable these reports.

Accessible, smartphone-enabled gait analysis with further validation may supplement or eventually reduce reliance on traditional assessments, which do not quantify impaired function or kinematic gait deviations; nor do they provide insights into the compensatory mechanisms associated with hemiparesis [63,64]. The 10mWT and 6MWT have been recommended as useful, psychometrically robust measures of mobility in neurological conditions [31], whereas the BBS, TUG, and 5xSTS may most accurately assess falls risk among community-dwelling older adults [29]. Gait velocity and other kinematic metrics have shown the ability to identify fallers [65,66]. Recent work suggests that wearable IMU/inertial sensor data, including accelerometry and variability indicators, improve fall prediction when combined with stopwatch-derived TUG duration [67,68]. Although this study did not capture falls, it is of note that both the app and therapist had 90–100% agreement regarding classification of fall risk (Table S4). This is notable when contemplating how mobile health technologies might facilitate remote evaluations comparably to standardized in-clinic assessments, as shown for the 6MWT self-administered by patients with cardiovascular disease [69,70], warranting future research in this area. Smartphones are also capable of collecting information about performance longitudinally across different real-life contexts and environments, leading to richer and more ecologically valid datasets than less regular testing that only occurs in location-based therapy settings. However, the value and feasibility of these data in post-stroke management should be further explored.

Previous work has validated IMUs/wearable sensors or smartphone algorithms against other laboratory devices and motion capture systems for outcome measurement [71,72,73,74], but there are fewer direct comparisons with the more typical administration protocols used clinically (i.e., therapist count or stopwatch timed). Additionally, this research is often conducted on healthy control subjects [75,76,77]. Systematic reviews have examined TUG in older adults [19] as well as a variety of smartphone-based mobility assessments for Parkinson’s Disease [78] and multiple sclerosis [79], concluding that current evidence supports this methodology. Relevant stroke research has focused on specific gait and balance analyses rather than the functional performance outcomes included here [80]. Uniquely, this study’s design involved comprehensive data collection by practicing therapists as part of an actual clinical program, and due to broad inclusion criteria, the patients with chronic stroke were diverse regarding physical ability. Thus, these results may have higher external validity for generalization to different implementation contexts.

Notable procedural considerations should be considered. Firstly, it is crucial that the smartphone has been securely positioned in a pocket or strap before each test to ensure accurate motion detection. At least one practice trial (or shorter ‘proxy’ for the 6MWT) could serve as both protocol familiarization for the subject and confirming successful data capture. This also allows a way to check for potential incorrect technique; for example, delayed start or partial repetitions in the CST, which is best avoided to maintain reproducibility. Other significant factors should be standardized within subjects, such as seat height, foot position, and the use of armrests [81], in particular when longitudinal analyses are conducted. For the 6MWT, calculating distance from lap counts does not incorporate turns and thus may tend to underestimate the total, especially if space limitations preclude longer walkways [82]. A maximum available 40-foot-long path was used here, but this logistical element likely contributed to the observed discrepancy between therapist and smartphone measurements. Therapist measurement of distance ambulated by counting laps of the 40 ft path may not account for additional steps taken and thereby distance ambulated during ambulatory turns; however, there was no independent distance reference to confirm this. The 6MWT outliers resulted from overestimation of stride length and irregular turning patterns that could not be fully analyzed. Despite these isolated or temporary issues, the algorithm demonstrated good fidelity. Therefore, an instrumented 6MWT may be preferable with the added benefit of obtaining spatiotemporal gait parameters from an application like OneStep; however, this requires further validation. The shorter Two-Minute Walk Test may further improve feasibility in restricted environments and when patients have less ambulation capacity [82]. Lastly, automated test administration (start/stop timer, etc.) frees the examiner up to focus on spotting/supervising a participant for safety.

This study has several limitations, including no a priori sample size calculation for the analyses conducted, potential bias from unblinded assessors, and lacking data to determine interrater reliability. It was not prospectively powered to detect specific outcomes and lacked a control group as the analysis was based on a convenience sample from a clinical program. The sample also included a heterogeneous population of participants with stroke, while consensus places the chronic phase of stroke at >6 months [83]; criteria for participation in the clinical program were >3 months based on established decreased expectation of spontaneous recovery [84]. Given the comprehensive nature of the clinical assessment, patient fatigue limited the collection of multiple trials of the 6MWT, which limited the ability to assess test–retest reliability of that measure. Future investigations must compare responsiveness or examine the relative longitudinal validity to determine which clinical variables best predict adverse events like falls, reflect therapeutic efficacy, and track clinically important change during recovery.

## 5. Conclusions

The associations observed between therapist- and smartphone-measured/administered outcomes underscore the potential of motion-sensing technology to efficiently enhance the objectivity of assessment in stroke rehabilitation utilizing TUG, 6MWT, and CST. The results suggest that the IMU-enabled smartphone applications, such as OneStep, can provide real-time, quantitative CST data that aligns closely with conventional counting, whereas TUG and 6MWT values require further evaluation. Within-session repeatability was not detectably different between the app and therapist for the CST and TUG measures; however, these findings are exploratory. Exploratory associations were also found when comparing patient-reported outcomes, gait metrics, and clinician-rated outcomes and remained significant even after controlling for multiple comparisons. Such automated techniques show promise to optimize routine practice and patient care across diverse clinical settings by providing objective impairment data. Confirmation requires future work with larger, prespecified studies that also address inter-rater reliability, as these findings are limited to within-session trial-to-trial repeatability and do not address between-day reliability, responsiveness to treatment, or longitudinal clinical utility.

## Figures and Tables

**Table 1:** Demographics and clinical characteristics of the study sample.

Variable	Count (Percentage), Mean (STD), or Median (Range)
Gender	21 male (50%), 21 female (50%)
Age	53.9 (13.2) years old
Height	66.6 (4.3) inches
Weight	167.6 (37.4) lbs.
Body Mass Index	26.4 (4.3) kg/m^2^
Time since CVA	2.5 (0.3–18.8) years
Stroke Type	22 ischemic (52%), 18 hemorrhagic (43%)
Stroke Location	20 left (48%), 22 right (52%)
Affected Side	22 left (52%), 20 right (48%)
Stroke Impact Scale—ADLs	37 (14–50)
Stroke Impact Scale—Mobility	36.5 (16–45)
Subjective Level of Recovery	47.3 (19.3)%
EuroQol-5D-5L VAS	59.3 (22.3)
EuroQol-5D-5L Mobility	3 (1–5)
EuroQol-5D-5L Usual Activities	3 (1–5)
Modified Rankin Scale	3 (1–4)
Barthel Index	95 (30–100)
NIH Stroke Scale	4 (0–12)
Berg Balance Scale	44.5 (3–54)
Five Times Sit to Stand	16.9 (6.2) s
10-Meter Walk Test Comfortable Gait Speed	0.57 (0.28) m/s
Fast Gait Speed	0.86 (0.41) m/s
Functional Gait Assessment	12.3 (7.1)

Abbreviations: CVA, cerebrovascular accident; ADLs, activities of daily living; VAS, visual analog scale.

**Table 2:** Participant and trial accounting for the paired therapist–app analyses.

Test	Participants with a Paired Value	Participants with Two/One Matched Valid Trial	Valid Trial Pairs, *n*	App Error, *n*/N (%)	Lost: App Error	Lost: App Value Missing	Lost: Not Recorded
CST	42 of 42	35/7	77	2/80 (2.5%)	0	0	0
TUG	39 of 42	34/5	73	3/76 (3.9%)	0	1	2
6MWT	31 of 42	single trial	31	5/36 (13.9%)	5	4	2

Averages for the CST and TUG are over trials with a valid therapist and app value (matched valid trial rule); the 6MWT was a single trial. App failures: *n*/N, where N is the number of trials (CST, TUG) or tests (6MWT) in which the app was run. Lost participants (no paired value): app error = the app reported a failure; app value missing = therapist value present but no app value; not recorded = no entry by either method.

**Table 3:** Agreement of therapist- and smartphone application-measured functional performance outcomes.

Outcome	*n*	Therapist Median (Range)	OneStep App Median (Range)	Mean Difference, Therapist—App (95% CI)	95% Limits of Agreement [95% CI Lower; Upper]	ICC(2,1) (95% CI)	Test of Systematic Difference	Paired d
CST (reps)	42	9.0 (4.0–21.5)	9.0 (4.0–21.5)	−0.08 (−0.26, 0.09)	−1.16 to 1.00 [−1.46, −0.87; 0.70, 1.29]	0.989 (0.980, 0.994)	Wilcoxon *p* = 0.399	−0.15
TUG (s)	39	15.5 (7.5–82.3)	18.5 (7.3–84.2)	−1.03 (−1.59, −0.47)	−4.39 to 2.33 [−5.35, −3.44; 1.37, 3.29]	0.995 (0.985, 0.998)	Wilcoxon *p* < 0.001	−0.60
6MWT (ft)	31	690 (110–1160)	731 (202–1223)	−69 (−110, −28)	−289 to 151 [−360, −218; 80, 222]	0.921 (0.776, 0.967)	T-test *p* = 0.002	−0.61

Difference = therapist—app. ICC(2,1): two-way random effects, absolute agreement, single measurement, with analytic 95% CI. Limits of agreement: mean difference ± 1.96 SD with the 95% CI of each limit in brackets. Systematic difference: Wilcoxon signed-rank test (CST, TUG; non-normally distributed differences) or paired t-test (6MWT); a non-significant *p*-value does not establish agreement. Paired Cohen’s d = mean difference/SD of the differences. ICC(1,1), ICC(3,1), ratio limits, and the literature thresholds are in Table S5.

**Table 4:** Within-session repeatability of the CST and TUG (trial 2 vs. trial 1, same session).

Test/Rater	*n*	Trial 1 Mean (SD) Trial 2 Mean (SD)	Trial 2—Trial 1 (95% CI) Trial-Order Test	ICC(2,1) (95% CI)	Spearman ρ (Association Only)	SEM	MDC95 (95% CI)	95% Limits of Agreement	Ratio Limits (Trial 2/Trial 1)	Heteroscedasticity ρ (Spearman |d| vs. Mean), *p*
CST OneStep app	35	9.2 (4.3) 9.6 (3.6)	0.46 (−0.46, 1.37) *p* = 0.020	0.775 (0.600, 0.879)	0.837	1.88	5.22 (2.69, 7.82) 56% of mean	−4.76 to 5.67	1.067 (0.720, 1.583) ^N/A^	ρ = 0.33 *p* = 0.050
CST Therapist	35	9.1 (4.5) 9.5 (3.6)	0.49 (−0.52, 1.49) *p* = 0.026	0.740 (0.545, 0.859)	0.794	2.07	5.73 (2.87, 8.63) 62% of mean	−5.25 to 6.22	1.078 (0.676, 1.718) ^N/A^	ρ = 0.29 *p* = 0.095
TUG OneStep app	34	25.6 (19.0) 24.1 (19.6)	−1.43 (−3.08, 0.22) *p* = 0.054	0.968 (0.936, 0.984)	0.960	3.45	9.55 (5.61, 13.36) 38% of mean	−10.70 to 7.84	0.934 (0.656, 1.329)	ρ = 0.66 *p* < 0.001
TUG Therapist	34	24.5 (18.3) 23.2 (19.3)	−1.28 (−2.46, −0.09) *p* = 0.006	0.982 (0.962, 0.991)	0.975	2.53	7.00 (4.86, 8.89) 29% of mean	−7.92 to 5.36	0.926 (0.752, 1.140)	ρ = 0.75 *p* < 0.001

Participants with two matched valid trials (the same participants for the app and the therapist); 1–5 min seated rest between trials. SEM = $\sqrt{\left(\mathrm{MSE}+(\mathrm{MSC}-\mathrm{MSE})/n\right)}$; MDC95 = 1.96 × $\sqrt{2\times \mathrm{SEM}}$, with bootstrap 95% CI and expressed as % of the mean. Trial order test: Wilcoxon signed-rank. Spearman’s rho measures association, not agreement, between the two trials and is shown for continuity only. Ratio limits (trial 2/trial 1, log scale) are the appropriate summary for TUG, where errors scale with magnitude (heteroscedasticity). For the CST, the heteroscedasticity test was borderline, so absolute 95% limits of agreement are used, and the ratio limits are marked not +applicable (^N/A^). Heteroscedasticity ρ is the Spearman correlation between the absolute trial-to-trial difference and the pair mean (Figure S5). The 6MWT was a single trial.

**Table 5:** Exploratory correlations of patient-reported outcomes with therapist- or smartphone application (‘app’)-measured clinical tests.

	BBS	5xSTS	10mWT-CGS	FGA	CST (App)	TUG (App)	6MWT (App)
SIS-ADLs	0.55 (0.25, 0.75) *p* < 0.001; q = 0.002 * *n* = 38	−0.54 (−0.75, −0.25) *p* < 0.001; q = 0.002 * *n* = 38	0.46 (0.14, 0.69) *p* = 0.004; q = 0.009 * *n* = 37	0.68 (0.43, 0.83) *p* < 0.001; q < 0.001 * *n* = 38	0.58 (0.30, 0.77) *p* < 0.001; q < 0.001 * *n* = 38	−0.56 (−0.76, −0.26) *p* < 0.001; q = 0.002 * *n* = 36	0.45 (0.09, 0.71) *p* = 0.013; q = 0.023 * *n* = 29
SIS mobility	0.44 (0.13, 0.68) *p* = 0.005; q = 0.010 * *n* = 38	−0.41 (−0.65, −0.09) *p* = 0.010; q = 0.018 * *n* = 38	0.51 (0.21, 0.73) *p* = 0.001; q = 0.003 * *n* = 37	0.54 (0.24, 0.74) *p* < 0.001; q = 0.002 * *n* = 38	0.38 (0.05, 0.63) *p* = 0.020; q = 0.031 * *n* = 38	−0.46 (−0.69, −0.13) *p* = 0.005; q = 0.010 * *n* = 36	0.48 (0.11, 0.73) *p* = 0.009; q = 0.016 * *n* = 29
SIS-9	0.18 (−0.15, 0.48) *p* = 0.278; q = 0.290 *n* = 38	−0.26 (−0.54, 0.07) *p* = 0.117; q = 0.137 *n* = 38	0.32 (−0.01, 0.59) *p* = 0.052; q = 0.071 *n* = 37	0.25 (−0.08, 0.53) *p* = 0.132; q = 0.147 *n* = 38	0.18 (−0.15, 0.47) *p* = 0.288; q = 0.291 *n* = 38	−0.33 (−0.60, 0.01) *p* = 0.052; q = 0.071 *n* = 36	0.51 (0.16, 0.75) *p* = 0.004; q = 0.009 * *n* = 29
EQ-5D-5L VAS	0.22 (−0.11, 0.51) *p* = 0.176; q = 0.187 *n* = 38	−0.34 (−0.60, −0.01) *p* = 0.039; q = 0.058 *n* = 38	0.28 (−0.06, 0.56) *p* = 0.094; q = 0.119 *n* = 37	0.27 (−0.06, 0.54) *p* = 0.106; q = 0.129 *n* = 38	0.25 (−0.08, 0.53) *p* = 0.136; q = 0.148 *n* = 38	−0.26 (−0.55, 0.08) *p* = 0.121; q = 0.138 *n* = 36	0.31 (−0.09, 0.61) *p* = 0.114; q = 0.136 *n* = 28
EQ-5D-5L mobility	−0.48 (−0.70, −0.18) *p* = 0.002; q = 0.005 * *n* = 39	0.38 (0.07, 0.63) *p* = 0.016; q = 0.026 * *n* = 39	−0.61 (−0.79, −0.34) *p* < 0.001; q < 0.001 * *n* = 38	−0.55 (−0.74, −0.26) *p* < 0.001; q = 0.002 * *n* = 39	−0.35 (−0.60, −0.03) *p* = 0.030; q = 0.046 * *n* = 39	0.61 (0.33, 0.79) *p* < 0.001; q < 0.001 * *n* = 37	−0.63 (−0.82, −0.31) *p* < 0.001; q = 0.002 * *n* = 29
EQ-5D-5L usual activities	−0.27 (−0.54, 0.05) *p* = 0.095; q = 0.119 *n* = 39	0.28 (−0.05, 0.55) *p* = 0.090; q = 0.019 *n* = 39	−0.39 (−0.64, −0.07) *p* = 0.016; q = 0.026 * *n* = 38	−0.32 (−0.58, 0.00) *p* = 0.045; q = 0.065 *n* = 39	−0.17 (−0.47, 0.15) *p* = 0.291; q = 0.291 *n* = 39	0.49 (0.18, 0.71) *p* = 0.002; q = 0.005 * *n* = 37	−0.54 (−0.77, −0.20) *p* = 0.002; q = 0.005 * *n* = 29
mRS	−0.67 (−0.82, −0.43) *p* < 0.001; q < 0.001 * *n* = 42	0.49 (0.21, 0.70) *p* < 0.001; q = 0.003 * *n* = 42	−0.53(−0.73, −0.24) *p* < 0.001; q = 0.002 * *n* = 40	−0.72 (−0.85, −0.50) *p* < 0.001; q < 0.001 * *n* = 42	−0.51(−0.72, −0.23) *p* < 0.001; q = 0.002 * *n* = 42	0.50 (0.20, 0.71) *p* = 0.001; q = 0.004 * *n* = 39	−0.54 (−0.76, −0.20) *p* = 0.002; q = 0.005 * *n* = 31

Values are Spearman’s rho correlation coefficients (lower, upper 95% confidence interval, Bonett–Wright), two-tailed raw *p*-value, and Benjamini–Hochberg false discovery rate adjusted q-value within the table (*, q < 0.05). The 6MWT (app) column uses the app distance as in Table 3.

**Table 6:** Exploratory association between therapist- and smartphone-measured clinical outcomes.

	Berg Balance Scale	Five Times Sit to Stand	10-Meter Walk Test-CGS	Functional Gait Assessment
Chair Stand Test (app)	0.59 (0.33, 0.77) *p* < 0.001; q < 0.001 * *n* = 42	−0.94 (−0.97, −0.88) *p* < 0.001; q < 0.001 * *n* = 42	0.43 (0.12, 0.66) *p* = 0.006; q = 0.006 * *n* = 40	0.57 (0.30, 0.76) *p* < 0.001; q < 0.001 * *n* = 42
Timed Up and Go (app)	−0.86 (−0.93, −0.72) *p* < 0.001; q < 0.001 * *n* = 39	0.57 (0.29, 0.76) *p* < 0.001; q < 0.001 * *n* = 39	−0.89 (−0.95, −0.78) *p* < 0.001; q < 0.001 * *n* = 39	−0.84 (−0.92, −0.69) *p* < 0.001; q < 0.001 * *n* = 39
Six-Minute Walk Test (app)	0.90 (0.78, 0.96) *p* < 0.001; q < 0.001 * *n* = 31	−0.55 (−0.77, −0.22) *p* = 0.001; q = 0.002 * *n* = 31	0.88 (0.74, 0.95) *p* < 0.001; q < 0.001 * *n* = 31	0.85 (0.68, 0.93) *p* < 0.001; q < 0.001 * *n* = 31

Values are Spearman’s rho correlation coefficients (lower, upper 95% confidence interval), two-tailed raw *p*-value and Benjamini–Hochberg adjusted q-value within the table (*, q < 0.05).

**Table 7:** Spatiotemporal parameters.

Gait Metric	Mean (STD) or Median (Range)
base width	16.28 (8.09–29.14) cm
cadence	85.58 (33.62–113.17) steps/min
cadence variability	2.51 (1.41–10.08) %
stride length	0.94 (0.22) m
velocity	0.68 (0.28) m/s
velocity variability	9.69 (5.62–19.85) %
step length asymmetry	4.39 (0.07–19.33) %
stance asymmetry	5.94 (0.34–46.53) %
double support	33.77 (25.69–59.47) %
double support asymmetry	1.66 (0.02–20.84) %
consistency	82 (0–95)
walk score	63 (44–84)

**Table 8:** Exploratory relationship between gait metrics and therapist- or smartphone application (‘app’)-measured functional performance outcomes.

	BBS	5xSTS	10mWT-CGS	FGA	30sCST (App)	TUG (App)
cadence variability	−0.74 (−0.87, −0.51) *p* < 0.001; q < 0.001 * *n* = 36	0.48 (0.16, 0.70) *p* = 0.003; q = 0.006 * *n* = 36	−0.74 (−0.87, −0.52) *p* < 0.001; q < 0.001 * *n* = 36	−0.77 (−0.89, −0.56) *p* < 0.001; q < 0.001 * *n* = 36	−0.46 (−0.69, −0.13) *p* = 0.005; q = 0.008 * *n* = 36	0.78 (0.57, 0.89) *p* < 0.001; q < 0.001 * *n* = 36
stride length	0.80 (0.61, 0.90) *p* < 0.001; q < 0.001 * *n* = 36	−0.34 (−0.61, 0.00) *p* = 0.044; q = 0.052 *n* = 36	0.74 (0.51, 0.87) *p* < 0.001; q < 0.001 * *n* = 36	0.76 (0.54, 0.88) *p* < 0.001; q < 0.001 * *n* = 36	0.40 (0.07, 0.65) *p* = 0.015; q = 0.020 * *n* = 36	−0.77 (−0.89, −0.56) *p* < 0.001; q < 0.001 * *n* = 36
velocity	0.82 (0.65, 0.91) *p* < 0.001; q < 0.001* *n* = 36	−0.42 (−0.67, −0.10) *p* = 0.010; q = 0.015 * *n* = 36	0.85 (0.70, 0.93) *p* < 0.001; q < 0.001 * *n* = 36	0.79 (0.59, 0.90) *p* < 0.001; q < 0.001 * *n* = 36	0.46 (0.14, 0.69) *p* = 0.005; q = 0.008 * *n* = 36	−0.90 (−0.95, −0.78) *p* < 0.001; q < 0.001 * *n* = 36
step length asymmetry	−0.47 (−0.70, −0.15) *p* = 0.004; q = 0.007 * *n* = 36	0.23 (−0.11, 0.52) *p* = 0.174; q = 0.188 *n* = 36	−0.41 (−0.66, −0.09) *p* = 0.012; q = 0.017 * *n* = 36	−0.38 (−0.64, −0.04) *p* = 0.023; q = 0.028 * *n* = 36	−0.23 (−0.52, 0.11) *p* = 0.182; q = 0.193 *n* = 36	0.42 (0.09, 0.66) *p* = 0.012; q = 0.017 * *n* = 36
stance asymmetry	−0.40 (−0.65, −0.07) *p* = 0.016; q = 0.021 * *n* = 36	0.16 (−0.18, 0.47) *p* = 0.349; q = 0.349 *n* = 36	−0.56 (−0.76, −0.26) *p* < 0.001; q < 0.001 * *n* = 36	−0.42 (−0.66, −0.09) *p* = 0.012; q = 0.017 * *n* = 36	−0.16 (−0.47, 0.18) *p* = 0.339; q = 0.345 *n* = 36	0.51 (0.19, 0.73) *p* = 0.002; q = 0.003 * *n* = 36
double support	−0.81 (−0.91, −0.62) *p* < 0.001; q < 0.001 * *n* = 36	0.39 (0.06, 0.65) *p* = 0.017; q = 0.022 * *n* = 36	−0.83 (−0.92, −0.66) *p* < 0.001; q < 0.001 * *n* = 36	−0.82 (−0.91, −0.64) *p* < 0.001; q < 0.001 * *n* = 36	−0.46 (−0.70, −0.14) *p* = 0.005; q = 0.008 * *n* = 36	0.84 (0.67, 0.92) *p* < 0.001; q < 0.001 * *n* = 36
double support asymmetry	−0.55 (−0.75, −0.24) *p* < 0.001; q = 0.001 * *n* = 36	0.18 (−0.16, 0.48) *p* = 0.295; q = 0.306 *n* = 36	−0.79 (−0.90, −0.58) *p* < 0.001; q < 0.001 * *n* = 36	−0.62 (−0.80, −0.34) *p* < 0.001; q < 0.001 * *n* = 36	−0.25 (−0.54, 0.09) *p* = 0.138; q = 0.152 *n* = 36	0.65 (0.38, 0.82) *p* < 0.001; q < 0.001 * *n* = 36
consistency	0.50 (0.19, 0.72) *p* = 0.002; q = 0.003 * *n* = 36	−0.30 (−0.58, 0.04) *p* = 0.073; q = 0.084 *n* = 36	0.37 (0.03, 0.63) *p* = 0.028; q = 0.034 * *n* = 36	0.57 (0.27, 0.76) *p* < 0.001; q < 0.001 * *n* = 36	0.28 (−0.06, 0.56) *p* = 0.098; q = 0.110 *n* = 36	−0.44 (−0.68, −0.12) *p* = 0.007; q = 0.011 * *n* = 36
walk score	0.83 (0.65, 0.92) *p* < 0.001; q < 0.001 * *n* = 36	−0.41 (−0.66, −0.08) *p* = 0.013; q = 0.017 * *n* = 36	0.86 (0.72, 0.94) *p* < 0.001; q < 0.001 * *n* = 36	0.81 (0.63, 0.91) *p* < 0.001; q < 0.001 * *n* = 36	0.44 (0.11, 0.68) *p* = 0.007; q = 0.011 * *n* = 36	−0.89 (−0.95, −0.78) *p* < 0.001; q < 0.001 * *n* = 36

Values are Spearman’s rho correlation coefficients (lower, upper 95% confidence intervals), two-tailed raw *p*-value and Benjamini–Hochberg adjusted q-value within the table (*, q < 0.05). Exploratory analyses without prespecified hypotheses. The 6MWT (app) column of the original table was removed: app 6MWT distance is calculated from the same recording and gait variables as these metrics, so those associations are shared-data associations, not independent evidence.

## Data Availability

The raw data supporting the conclusions of this article will be made available by the authors upon request.

## Supplementary Materials

The following supporting information can be downloaded at: https://www.mdpi.com/article/10.3390/s26216947/s1, Figure S1: Data availability by participant: Figure S2: Within-session repeatability of the therapist-measured functional outcomes; Figure S3: Leave-one-participant-out ICC(2,1) for the agreement analyses; Figure S4: Leave-one-therapist-out ICC(2,1) for the agreement analyses; Figure S5: Within-session repeatability by participant (matched participants); Table S1: Difference between therapist and app measurements (therapist-app) by therapist; Table S2: Outlier sensitivity analyses of agreement and within-session repeatability; Table S3: Multiplicity control and outlier sensitivity of the exploratory correlations; Table S4: Paired fall risk classification agreement (therapist vs. app); Table S5: Additional agreement statistics and literature thresholds; Table S6: Participants with and without a paired 6MWT; Table S7: Leave-one-therapist-out sensitivity of the therapist vs app agreement analysis.
