# Supplementary material for: Validity of Functional Performance Outcomes and Gait Metrics Measured by Smartphone Application in Patients with Chronic Stroke
Source: Sensors (Basel). 2026 Nov 3;26(21):6947. doi: 10.3390/s26216947 (PMC13766453; doi:10.3390/s26216947)
Supplement: Supplementary file 1 [file sensors-26-06947-s001.zip › sensors-4552740-supplementary.pdf]

Supplementary Materials

The following supporting information accompanies the article: Tables S1–S6 and Figures S1–S3.

Table S1. Difference between therapist and app measurements (therapist—app) by therapist.

Test	Therapist	n	Mean difference (therapist—app)	SD
CST	one	31	−0.16	0.60
CST	four	2	0.00	0.00
CST	two	5	0.20	0.45
CST	three	4	0.12	0.25
CST	Kruskal–Wallis			p = 0.258
TUG	one	29	−1.25	1.86
TUG	four	1	−0.62	
TUG	two	5	−0.24	1.41
TUG	three	4	−0.52	0.57
TUG	Kruskal–Wallis			p = 0.438
6MWT	one	25	−65	115
6MWT	two	4	−49	91
6MWT	three	2	−151	133
6MWT	Kruskal–Wallis			p = 0.653

Therapist one assessed most participants; no participant was assessed by more than one therapist. Kruskal–Wallis test across therapists (exploratory; three of four therapists assessed six or fewer participants).

Table S2. Outlier sensitivity analyses of agreement and within-session repeatability.

Analysis	Test	Scenario	n	ICC(2,1)	Mean difference	Limits of agreement/MDC95
Agreement	CST	All data	42 (0 removed)	0.989	−0.08	−1.16 to 1.00
Agreement	CST	Flagged outliers excluded	40 (2 removed)	0.998	0.03	−0.47 to 0.52
Agreement	CST	Extreme outliers excluded	42 (0 removed)	0.989	−0.08	−1.16 to 1.00
Agreement	CST	Strap-worn participants (records 6, 33) excluded	40 (2 removed)	0.994	−0.03	−0.82 to 0.77
Agreement	TUG	All data	39 (0 removed)	0.995	−1.03	−4.39 to 2.33
Agreement	TUG	Flagged outliers excluded	32 (7 removed)	0.999	−0.64	−1.70 to 0.42
Agreement	TUG	Extreme outliers excluded	36 (3 removed)	0.999	−0.64	−2.20 to 0.93
Agreement	TUG	Strap-worn participants (records 6, 33) excluded	37 (2 removed)	0.995	−1.00	−4.42 to 2.42
Agreement	6MWT	All data	31 (0 removed)	0.921	−68.80	−288.55 to 150.95

Agreement	6MWT	Flagged outliers excluded	30 (1 removed)	0.941	−58.04	−246.98 to 130.89
Agreement	6MWT	Extreme outliers excluded	31 (0 removed)	0.921	−68.80	−288.55 to 150.95
Agreement	6MWT	Strap-worn participants (records 6, 33) excluded	29 (2 removed)	0.921	−69.34	−295.46 to 156.78
Repeatability	CST app	All data	35 (0 removed)	0.775	0.46	MDC95 5.22
Repeatability	CST app	Flagged outliers excluded	27 (8 removed)	0.927	0.56	MDC95 1.81
Repeatability	CST app	Extreme outliers excluded	33 (2 removed)	0.893	0.70	MDC95 3.03
Repeatability	CST app	Strap-worn participants (records 6, 33) excluded	33 (2 removed)	0.790	0.27	MDC95 5.10
Repeatability	CST therapist	All data	35 (0 removed)	0.740	0.49	MDC95 5.73
Repeatability	CST therapist	Flagged outliers excluded	27 (8 removed)	0.927	0.52	MDC95 1.77
Repeatability	CST therapist	Extreme outliers excluded	32 (3 removed)	0.905	0.59	MDC95 2.88
Repeatability	CST therapist	Strap-worn participants (records 6, 33) excluded	33 (2 removed)	0.777	0.21	MDC95 5.36
Repeatability	TUG app	All data	34 (0 removed)	0.968	−1.43	MDC95 9.55
Repeatability	TUG app	Flagged outliers excluded	31 (3 removed)	0.986	−0.83	MDC95 6.66
Repeatability	TUG app	Extreme outliers excluded	33 (1 removed)	0.982	−0.90	MDC95 7.24
Repeatability	TUG app	Strap-worn participants (records 6, 33) excluded	32 (2 removed)	0.967	−1.41	MDC95 9.80
Repeatability	TUG therapist	All data	34 (0 removed)	0.982	−1.28	MDC95 7.00
Repeatability	TUG therapist	Flagged outliers excluded	31 (3 removed)	0.987	−0.82	MDC95 6.24
Repeatability	TUG therapist	Extreme outliers excluded	34 (0 removed)	0.982	−1.28	MDC95 7.00
Repeatability	TUG therapist	Strap-worn participants (records 6, 33) excluded	32 (2 removed)	0.982	−1.23	MDC95 7.13

Flagged outliers: outside Tukey's fences (1.5 IQR) applied to the paired differences (log ratio when errors scaled with magnitude); extreme: 3 IQR. Because outliers are defined from the differences, exclusion improves agreement by construction; read as best-case estimates. Strap-worn: the two participants (records 6 and 33) who wore the device on a strap rather than in a pocket; this exclusion is unrelated to the size of the differences.

Table S3. Multiplicity control and outlier sensitivity of the exploratory correlations.

Original table	Tests	Raw p<0.05	BH q<0.05	Holm <0.05	BH across tables <0.05	FDR conclusion changes when outliers removed	Sign flips	FDR conclusion changes when strap-worn participants (records 6, 33) removed
Table 5	49	34	32	17	32	6/49	1	3/49
Table 6	12	12	12	12	12	0/12	0	0/12
Table 8	54	46	45	28	45	7/54	0	0/54

BH, Benjamini–Hochberg false discovery rate procedure (q < 0.05) within each original table; Holm, Holm's procedure; BH across tables adjusts all tests together. Pairs of the app 6MWT with the gait metrics (n = 9) are excluded.

Table S4. Paired fall risk classification agreement (therapist vs. app).

Test	Cutoff	n	At risk: therapist/app	Both/therapist only/app only/neither	Agreement	Cohen's kappa	PABAK
CST	below age/sex norm (Rikli & Jones 1999/CDC STEADI)	15	15/15	15/0/0/0	100%	not estimable (expected agreement = 1.00)	1.00
TUG	>=14 s (Andersson 2006)	39	23/25	23/0/2/14	95%	0.89[0.75, 1.00]	0.90
6MWT	<331.65 m (Regan 2020)	31	29/28	27/2/1/1	90%	0.35 [-0.35, 1.00]	0.81

Agreement of two measurements against a literature threshold; not validation against actual falls (no fall data were collected). CST norms apply to participants aged 60-94 years. Kappa is not estimable for the CST because all participants were classified as at risk by both methods. For the 6MWT, most participants were classified as at risk by both raters, so kappa is low relative to percent agreement, and its 95% CI is wide. Kappa CIs in this table are analytic; bootstrap CIs (2,000 resamples) are given in the Results. PABAK, prevalence-adjusted bias-adjusted kappa.

Table S5. Additional agreement statistics and literature thresholds.

Test	ICC(1,1)	ICC(3,1)	Ratio limits (therapist/app)	Prop. bias p	Pearson r/Spearman ϱ	Literature threshold	|Differences| within threshold	Limits vs threshold
CST	0.989 (0.980, 0.994)	0.989 (0.980, 0.994)	0.990 (0.863-1.137)	0.608	0.989/0.980	MDC 2 reps	±2.0 reps: 98%	±2.0 reps: inside
TUG	0.995 (0.990, 0.997)	0.996 (0.993, 0.998)	0.949 (0.795-1.134)	0.264	0.996/0.990	MDC 3.2 s	±3.2 s: 92%	±3.2 s: outside
6MWT	0.920 (0.842, 0.961)	0.940 (0.879, 0.971)	0.850 (0.558-1.296)	0.708	0.940/0.926	MDC 13%	±13.0%: 45%	±13.0%: outside

ICC(1,1), one-way random effects; ICC(3,1), two-way mixed effects, consistency. Pearson r and Spearman ϱ describe association only.

Table S6. Participants with and without a paired 6MWT.

Characteristic	Paired 6MWT (n=31) median (IQR)	No paired 6MWT (n=11) median (IQR)	p
Age, Years	53.0 (46.0-61.5)	58.0 (46.0-69.5)	0.352
Time since Stroke, Years	2.7 (1.0-5.7)	2.0 (0.7-3.0)	0.440
Modified Rankin Scale	3 (2-3)	3 (2-4)	0.531
Berg Balance Scale	44.0 (33.0-50.0)	45.0 (29.0-49.0)	0.785

Functional Gait Assessment	12.0 (8.0-15.5)	12.0 (6.5-17.0)	0.977
10 m Walk, Comfortable Speed, m/s	0.57 (0.38-0.77)	0.52 (0.35-0.82)	0.674
Five Times Sit to Stand, s	15.1 (12.4-18.2)	17.1 (15.3-23.7)	0.097
TUG, Therapist, s	15.5 (11.4-36.5)	18.5 (12.1-31.9)	0.578
CST, Therapist, reps	10.0 (7.8-11.5)	8.5 (6.2-9.2)	0.140
EQ-5D-5L Mobility (1-5)	3 (2-4)	3 (2-3)	0.987
Male Sex, n (%)	14 (45%)	7 (64%)	0.484

Mann–Whitney U test (continuous and ordinal variables) or Fisher's exact test (sex).

Table S7. Leave-one-therapist-out sensitivity of the therapist vs app agreement analysis.

Test	Therapist excluded	n excluded	n remaining	ICC(2,1) (95% CI)	Mean difference (95% CI)
CST	(all data)	0	42	0.989 (0.980, 0.994)	−0.08 (−0.26, 0.09)
CST	four	2	40	0.989 (0.979, 0.994)	−0.09 (−0.27, 0.09)
CST	one	31	11	0.997 (0.989, 0.999)	0.14 (−0.08, 0.35)
CST	three	4	38	0.984 (0.970, 0.992)	−0.11 (−0.29, 0.08)
CST	two	5	37	0.989 (0.980, 0.995)	−0.12 (−0.31, 0.06)
TUG	(all data)	0	39	0.995 (0.985, 0.998)	−1.03 (−1.59, −0.47)
TUG	four	1	38	0.995 (0.985, 0.998)	−1.04 (−1.61, −0.47)
TUG	one	29	10	0.998 (0.992, 0.999)	−0.39 (−1.11, 0.33)
TUG	three	4	35	0.994 (0.983, 0.998)	−1.09 (−1.71, −0.47)
TUG	two	5	34	0.995 (0.983, 0.998)	−1.15 (−1.76, −0.54)
6MWT	(all data)	0	31	0.921 (0.776, 0.967)	−68.80 (−109.93, −27.68)
6MWT	one	25	6	not estimable (n remaining < 8)	
6MWT	three	2	29	0.927 (0.805, 0.969)	−63.15 (−105.38, −20.92)
6MWT	two	4	27	0.917 (0.758, 0.966)	−71.69 (−117.63, −25.75)

Each row drops every participant assessed by one therapist and recomputes ICC(2,1) and the mean difference (therapist—app) on the remainder; 95% CIs are analytic (Shrout & Fleiss 1979; McGraw & Wong 1996). Excluding the therapist who assessed most participants removed most of the sample, substantially widening the uncertainty and, for the 6MWT, leaving too few participants (n<8) to estimate agreement at all.

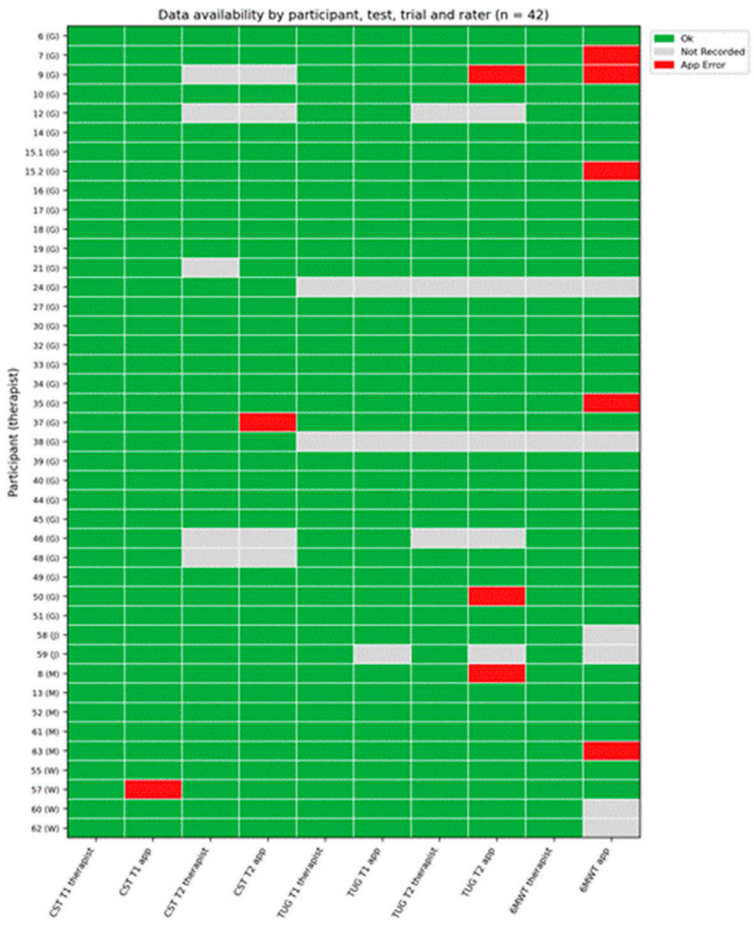

Figure S1. Data availability by participant, test, trial, and rater (green, valid; red, app error; gray, not recorded). Participants are grouped by therapist.

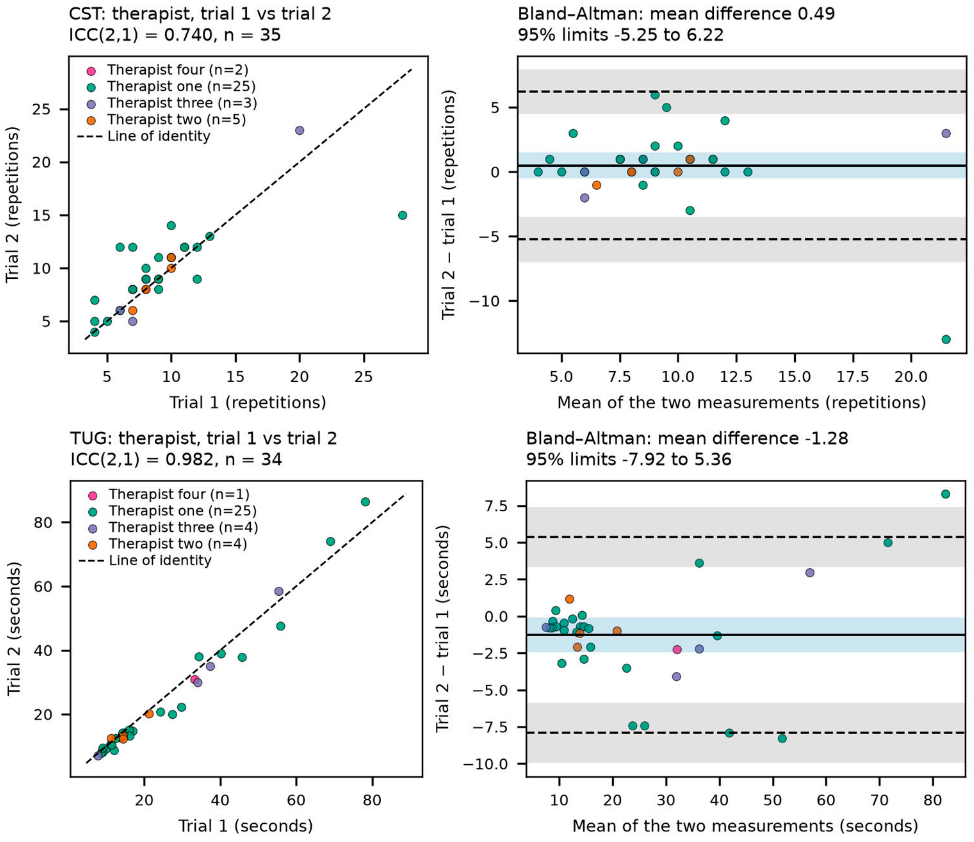

Figure S2. Within-session repeatability of the therapist-measured Chair Stand Test (top) and Timed Up and Go (bottom), trial 2 vs. trial 1. The panels are the same as in Figure 2.

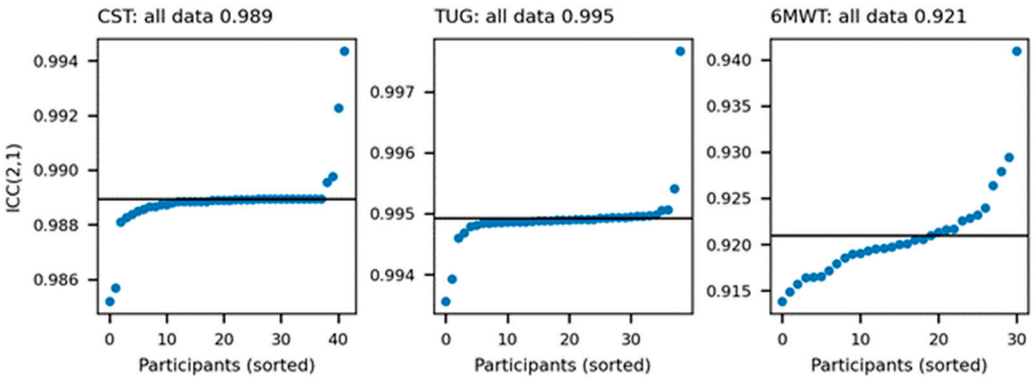

Figure S3. Leave-one-participant-out ICC(2,1) for the agreement analyses (horizontal line, all data).

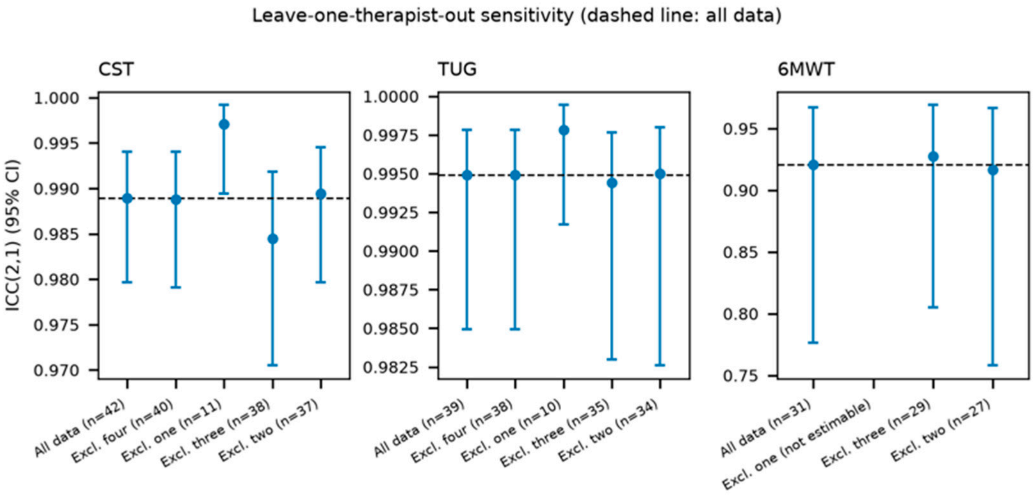

Figure S4. Leave-one-therapist-out ICC(2,1) for the agreement analyses, with 95% CIs (dashed line, all data).

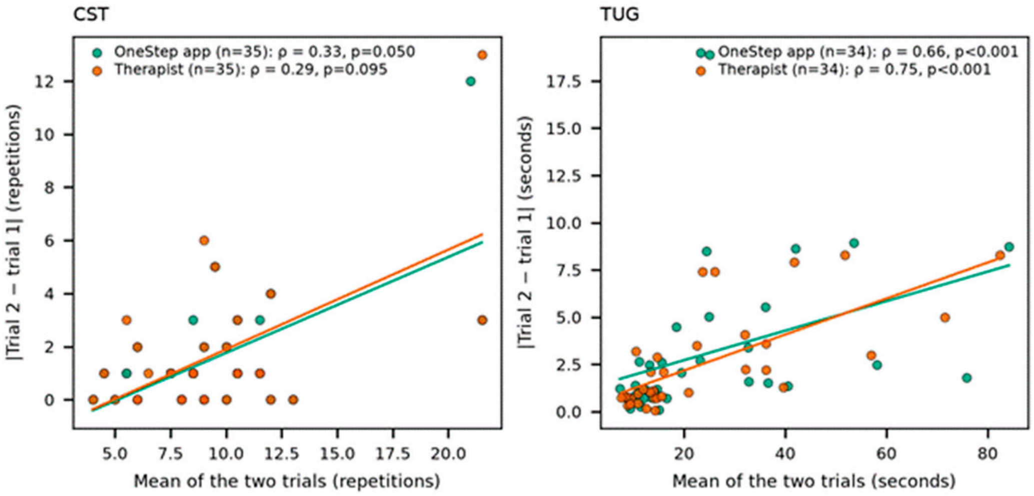

Figure S5. Within-session repeatability by participant (matched participants): absolute trial 2—trial 1 difference against the mean of the two trials, for the

OneStep app and the therapist, with least-squares lines. ϱ is the Spearman correlation between the absolute difference and the mean (heteroscedasticity test, as in Table 4); $\varrho > 0$ indicates that trial-to-trial differences grew with the mean.
